# Topics of Public Interest

**Published:** 1905-03

**Authors:** 


### TOPICS OF PUBLIC INTEREST.

### Civil Service Examinations for the State and County Service.

The State Civil Service Commission announces general examinations to be held March 11, 1905, including among others the following positions: assistant in geology, State Museum; bridge draughtsman, State Engineer's office; fireman; foreman of broom shop, state prisons; junior bridge draughtsman, State Engineer's office; matron, state hospitals for the insane; orderly (male nurse), Erie County Hospital; steward (woman), institutions for women; supervisor of farm cottage, Rochester State Industrial School; taxidermist, State Museum; veal inspector; woman physician, state hospitals and institutions.

Applications for these examinations must be made on or before March 7. Full particulars of the examination and blank applications may be obtained by addressing the chief examiner of the Commission at Albany.

### Army Medical Corps Examinations.

Preliminary examinations for appointment of assistant surgeons in the army will be held on May 1 and August 1, 1905, at points to be hereafter designated. Permission to appear for examination can be obtained upon application to the Surgeon General, U. S. Army, Washington, D. C., and from whom full information concerning the examination can be procured.

The examinations will be held concurrently throughout the country at points where boards can be convened. Due consideration will be given to the localities from which applications are received, in order to lessen the traveling expenses of applicants as much as possible.

For the examinations of May 1, applications must be complete and in possession of the Surgeon General on or before April 1, and for the examination of August 1, on or before July 1.

---

A new charter of the American National Red Cross has been passed by both houses of Congress. This charter, which puts the organisation practically under the control of the government, was drawn by two former secretaries of state—Mr. Richard Olney and Mr. John W. Foster.—*Med. Age.*

